# First contact: Fine structure of the impact flash and ejecta during hypervelocity impact

**DOI:** 10.1093/pnasnexus/pgad214

**Published:** 2023-07-11

**Authors:** Gary Simpson, Justin Moreno, Matthew Shaeffer, K T Ramesh

**Affiliations:** Mechanical Engineering, Johns Hopkins University, Baltimore, 21218 MD, USA; Hopkins Extreme Materials Institute, Johns Hopkins University, 21218 MD, USA; Hopkins Extreme Materials Institute, Johns Hopkins University, 21218 MD, USA; Hopkins Extreme Materials Institute, Johns Hopkins University, 21218 MD, USA; Mechanical Engineering, Johns Hopkins University, Baltimore, 21218 MD, USA; Hopkins Extreme Materials Institute, Johns Hopkins University, 21218 MD, USA

## Abstract

Hypervelocity impacts are a significant threat in low-earth orbit and in hypersonic flight applications. The earliest observable phenomena and mechanisms activated under these extreme conditions are typically obscured by a very bright flash, called the impact flash, that contains the signatures of the critical mechanisms, the impacting materials, and the impact environment. However, these signatures have been very difficult to observe because of the small length and time scales involved coupled with the high intensities associated with the flash. Here we perform experiments investigating the structure and characteristics of the impact flash generated by 3 km s${}^{-1}$ spherical projectile impacts on structural metals using temporally co-registered high-resolution diagnostics. Reciprocal impact configurations, in which the projectile and target material are swapped, are used to demonstrate the coupling of early-stage mechanisms in the flash and later-stage ejection mechanisms responsible for the development of the impact crater.

**Significance Statement**

The very first instants of a hypervelocity impact event develop the most extreme conditions experienced by structural materials. Such high-velocity impacts are a limiting factor in a number of aerospace and planetary applications, including spacecraft protection and hypersonic flight through the lower atmosphere. The mechanisms activated under these extreme conditions are typically obscured by a very bright flash, called the impact flash, that contains within it the signatures of the critical mechanisms, the impacting materials, and the impact environment. Our observations demonstrate that impact jetting, ablation, and other atmospheric interactions are critical mechanisms in rarefied ambient gas environments.

## Introduction

**Fig. 1.**
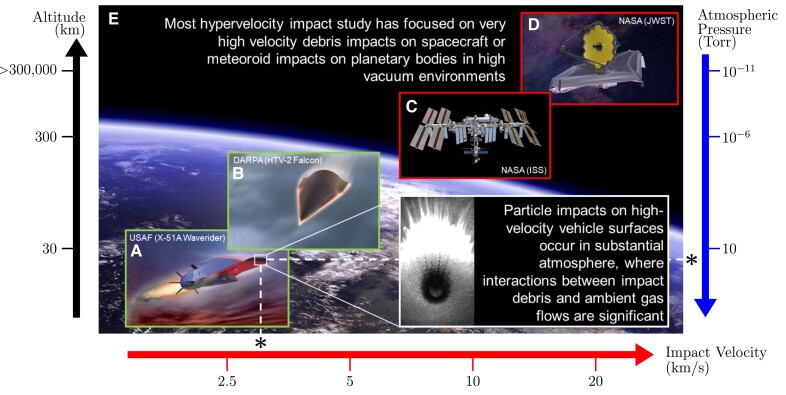
Hypervelocity impacts occur in a wide range of aerospace and planetary applications, with a range of impact velocities and ambient atmospheres. The experimental conditions investigated in this work, for example, are equivalent to the ambient atmospheric density at an altitude of approximately 30 km (relevant to the upper and lower operational envelopes of high-velocity air-breathing and boost-glide vehicles respectively). Background images: A) https://airandspace.si.edu/multimedia-gallery/100520-f-9999b-111jpg, B) https://cdn.mos.cms.futurecdn.net/q6L8JJJgz7TdtQEgYywEYU-1200-80.jpg, C) https://blogs.nasa.gov/spacestation/wp-content/uploads/sites/240/2021/12/blog_jsc2021e064216_alt.jpg, D) https://www.nasa.gov/sites/default/files/thumbnails/image/16658886757_69219ed7f8_k.jpg, E) https://ualr.edu/geology/files/2006/10/EarthHorizon.png.

High-velocity impact occurs in critical applications ranging from supersonic aircraft transiting dusty atmospheres to micrometeoroids impacting orbiting satellites (Fig. 1). Such impacts often generate a short but intense emission of electromagnetic (EM) radiation that is referred to as the impact flash. The wavelengths in the flash can range from radio frequencies (RF) to ultraviolet (UV). Both wavelength and intensity vary greatly with impact conditions and environment, indicating a coupling between the impact-induced mechanisms and the EM signature. Impact flash phenomena are observed across a wide-range of conditions. For the last couple decades, impact flash has been successfully used to monitor ultra-high velocity (>20 km s${}^{-1}$) lunar meteoroid impacts allowing better measurements of meteoroid flux in our space environment (1–3). However, we also observe surprisingly intense flash phenomena in much lower velocity (<5 km s${}^{-1}$) laboratory experiments despite massive differences in involved energies, materials, and ambient conditions. Experimentally, the intense flash often prevents optical observation of the earliest moments of projectile contact and target penetration in laboratory conditions. However, the flash also contains important information on the highest energy processes occurring during first contact, and understanding this signature is very valuable in planetary, aerospace, and national security applications.

The earliest phase (first contact) of projectile/target interaction in hypervelocity impacts is extremely difficult to access with time-resolved in-situ methods. The connections between early mechanisms (such as impact jetting) and the later-stage mechanisms of deformation and failure have therefore been difficult to determine. For the purposes of this manuscript, we define the early phase mechanisms as those occurring within the time-scale $\tau =\frac{V_{0}}{r_{i}}$, where $V_{0}$ is the impact velocity magnitude and $r_{i}$ is the radius of the spherical impactor. We further define late-stage mechanisms as those developed at times greater than 5τ. A better understanding of how the initial impact mechanisms evolve and then connect to the late-stage mechanisms responsible for crater growth and large-scale material ejection is critical to high-velocity applications. For example, at hypersonic flow velocities (Mach number M≥5) particle impacts can create substantial damage, alter surface characteristics, and create energetic mixed-phase flows within the boundary layer, degrading control surfaces and disrupting critical aerodynamic gas flows near the impacted surface (4). Here we use direct time-resolved and in situ experimental methods to examine both the early-stage and later-stage mechanisms.

Prior experimental studies of high-velocity impact flash revealed a variety of behaviors, (5–9) in terms of the evolution of the total flash intensity with time. At high impact velocities, the history of the flash intensity shows two (8, 10) or three (9, 11) regimes: an initial sub-microsecond duration “spike” in intensity (often attributed to impact jetting (8, 11–13)), a long duration “tail” (previously attributed to ejecta ablation (11, 14)), and occasionally an intermediate peak (attributed to shock-induced vaporization in the crater (11)).

Material–atmosphere interactions also have dramatic effects on the impact flash. (15, 16) Laboratory experiments have shown overall flash luminosity—the long-duration “hump-like” luminosity peak in particular—to be highly sensitive to ambient atmospheric pressure (9), intensifying in amplitude and decreasing in duration with higher atmospheric pressure. This peak persists, albeit at reduced intensity, at much higher vacuum (<75 mTorr) than the present work investigates, but there are no experimental launch facilities capable of performing these scale of impacts in any kind of atmosphere approaching a true high-vacuum environment where the potential effects of ambient atmosphere would be completely eliminated (9, 11).

A number of experimental investigations of the spectral content of the impact flash have been performed (13, 15, 17–19) at high impact velocities (5–7 km $s^{-1}$). At impact velocities in excess of 10–15 km s${}^{-1}$ projectiles may be shock vaporized entirely, and the radiation dynamics and expansion of the vapor and plasma dominate the impact flash (20, 21). The study of orbital and planetary impacts being a common motivation for such work, laboratory hypervelocity experiments often utilize low-density and lower strength materials to more closely resemble the dynamics of real-life impacts despite the velocity limitations of launch systems. Kadono and Fujiwara (22) imaged the expansion of impact vapor clouds under such conditions, using nylon projectiles (which vaporize at lower velocities). Other works have examined the chemistry and evolution of impact vapor clouds (10, 23–26) at late times by combining imaging and spectroscopy. Mihaly et al. (24, 25) investigated impact flash using nylon cylinders impacting aluminum targets at approximately 6 km s${}^{-1}$, conditions in which near total vaporization of the projectile would be expected, (22) but their experimental configuration prevented imaging, spectroscopic, or photometric observations before 3 μs after initial impact (equivalent to ∼20τ).

In the extreme case of the aforementioned lunar impacts, meteoroid impact velocities exceeding 20 km s${}^{-1}$ are commonplace depending upon the meteoroid source (27, 28); the massive specific kinetic energy of these impacts produce large-scale vaporization and ionization. At laboratory impact speeds of ≤5 km s${}^{-1}$ with metals, there is typically insufficient energy to directly vaporize engineering materials. However, strong atomic and ionic gas phase characteristic radiation is still observed at modest impact velocity, indicating that vaporization of projectile and target material is occurring. The question then becomes not why flash phenomena are seen in high-energy impacts (e.g. lunar impacts) but why intense impact flashes are seen in relatively low-energy laboratory impacts. Here we investigate the flash behavior at such impact speeds in structural metals, examining the early-stage mechanisms of projectile–target interaction and their role in the production of energetic, radiating material. We also investigate the evolution of the early phase ejection and flash phenomena and the mechanistic transition to the later-stage development of the familiar and easily resolved ejecta cone. We provide direct experimental observations of impact jetting and flash behavior in reciprocal projectile/target systems (necessary to separate material and geometry effects on the jetting mechanism). Impact jetting (12, 29, 30) has also received considerable attention in the planetary science community, as both a viable melt and vapor generating mechanism active across impact scales (31).

## Results

**Fig. 2.**
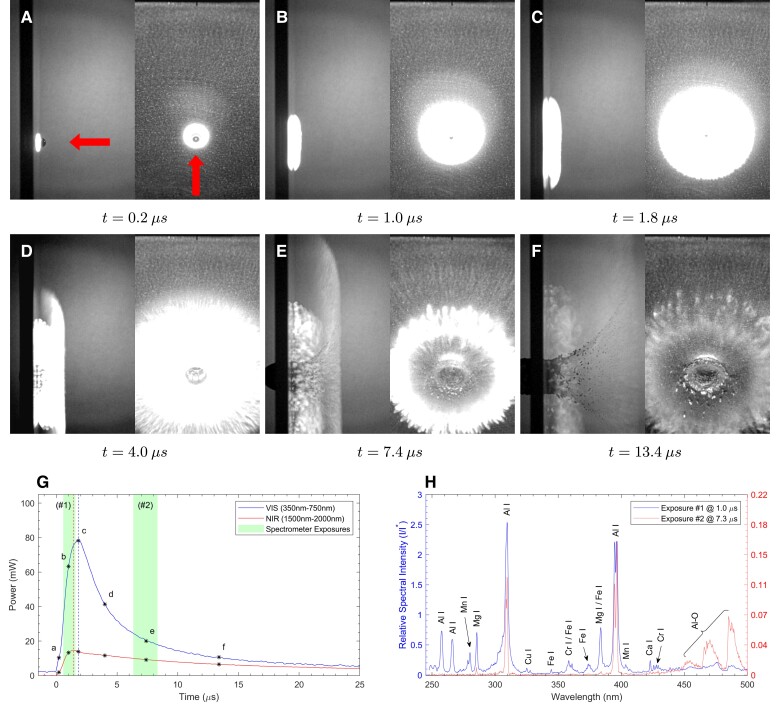
304SS projectile impacting 6061Al target at 90${}^{\circ }$ obliquity with normal velocity of 3.0 km s${}^{-1}$ in 15 Torr air. Selected images, A–F) are shown as time-matched pairs with both side camera (left image) and front camera (right image) views. Exposure time is 110ns for all examples presented. Visual (VIS, 350–750 nm) and near-infrared (NIR, 1,500–2,000 nm) luminosities are shown in G); peak luminosities are indicated by vertical dashed lines. Image times corresponding to A–F) are indicated on G), and spectrometer exposure windows for each experiment are shaded. The collected spectra are shown in H), where the spectral intensity I is normalized with respect to I*, the magnitude of the 396.1 nm Al I line of the Al/Al-90${}^{\circ }$ configuration shown in Fig. 4. As such, all spectral intensities are plotted relative to the intensity of this peak in the symmetric, Al/Al impact at normal incidence and the magnitudes of any spectral peak are read in comparison to that experiment. The first spectrometer exposure (#1) is measured with respect to the left-hand scale of H); the second spectrometer exposure (#2)—which is generally much dimmer than the first and has been rescaled to visually match both 396.1 nm Al I peaks and make spectral shape differences easier to see—should be measured with respect to the right-hand scale. For instance, in H) it can be observed that the 396.1 nm Al I peak relative spectral intensity (I/I*) in the first exposure (#1) is 2.2 and the second exposure is (#2) 0.16. So the early exposure 396.1 nm Al emission peak for this experiment is over twice as intense as the Al/Al-90${}^{\circ }$ impact, whereas the same peak has diminished in intensity by a factor of almost 14 by the late exposure.

The impact of a 5 mm diameter sphere made of 304 stainless steel (304SS) into a 6061-T6 Al alloy target plate at a normal impact velocity of 3 km s${}^{-1}$ is shown in Fig. 2. In this case, the characteristic timescale is τ=0.8μs=800ns. Fig. 2A to F shows a selection of high-speed camera images taken over the first few μs after impact, with each image pair showing the side view on the left and the front view on the right. The impact flash is first observed as an intensely bright disk within 200 ns after contact (Fig. 2A), with the impactor still visible. The bright disk-shaped zone grows over the next 800 ns, and begins to show (Fig. 2B) some structure. The center of the impact crater is just visible in the front view of Fig. 2C. In Fig. 2D, the side view of the bright zone shows two distinct components, a bright inner zone and a faster-moving, less intense outer zone. The component farther away from the target plate is expanding (radially) faster than is the inner component. At this time, the radial periphery of the outer component shows signs of radial structure. Just over 3 μs later, looking at the side view of Fig. 2E, the outer component shows all of the characteristics of the classical ejecta cone, with dark ejecta particles in the cone; the inner component now shows perceptible bright blobs, while the outer component is now difficult to see in the front view. The outer parts of the darker ejecta cone smoothly transition into the edge of the outer flash zone, suggesting trajectory continuity between the ejected material forming the outer flash boundary and the excavated solid material forming the dark ejecta cone. By the time of Fig. 2F, 13.4 μs after impact, the inner component is cooling and dissipating with radial fingers propagating slowly at the radial edge. The outer emission zone has dimmed as well, and is just perceptible towards the right edge of the image frame; the full extent of the ejecta cone is now visible. Note that the target plate has been fully perforated at this time.

The outer emission zone in Fig. 2D to F is brightest along the uprange direction and very faint along the target plate surface as it expands. The streak-like appearance of the outer zone front in Fig. 2D and E indicates that this zone contains condensed particles. In contrast, the inner emission zone in Fig. 2D to F is bright both along the target surface and along the impact direction (uprange), consistent with an expanding vapor cloud (e.g. (22)). The front view in Fig. 2E shows that the circumference of the inner zone has now developed radial finger-like structures, instabilities resulting from the interaction of the vapor cloud with the ambient atmosphere (32). The corresponding evolution of the visual (VIS) and near-infrared (NIR) luminosities are shown in Fig. 2G, with the times corresponding to each of the high-speed camera images noted in Fig. 2A to F. The peak in NIR intensity occurs just before the time corresponding to Fig. 2C, while the VIS luminosity peaks at 1.8 μs, corresponding to Fig. 2C. Both curves decay thereafter over about 15 μs, during the times corresponding to Fig. 2D to F. The luminosity data are spatially integrated over a larger field of view than shown in these images, and so the observed decay is not due to material leaving the field of view. The cavity generated by the impact no longer expands after about 10 μs post impact (i.e. new ejecta are not being added after this time).

Fig. 2H shows short exposure UV/VIS gated spectra obtained during the flash event; two separate spectra are obtained over the time-windows (exposures) marked #1 and #2 in Fig. 2G (one obtained just before the VIS luminosity peak, and the other well after). Both spectra are entirely dominated by characteristic (line) emission. The most intense characteristic emissions observed are Al I atomic peaks (33) as indicated on the spectra, as are Mg and Cu emissions (chief alloying elements in 6061Al). Fe and Cr species, from the 304SS projectile, can also be seen in spectrum #1, but are largely absent at the later time. The magnitude of all observable atomic peaks are reduced by at least an order of magnitude in the later-time spectrum. Significant Al-O molecular band emission (25) is present in spectrum #2, indicating the vapor-phase combustion of Al with ambient oxygen within the first 10 μs of the impact flash. While the overall intensity decays from #1 to #2 as indicated by the numerical peak magnitudes, the Al-O emission is the only component to increase relative to the atomic emission. The two spectra show that the significant combustion only occurs well after the luminosity peaks, demonstrating that combustion is not the chief cause of the light emitted during the times up to the peaks of the VIS and IR components of the flash. Thus the vaporized material is hot and radiating well before significant combustion occurs. Combustion emission was also observed at late times by Tandy et al. (25) during the high-speed impact of nylon projectiles on aluminum plate in 1 Torr air. Oblique impacts for this projectile and target material combination and others are discussed in Supplementary Section S4 and Supplementary Figs. S4–S6.

**Fig. 3.**
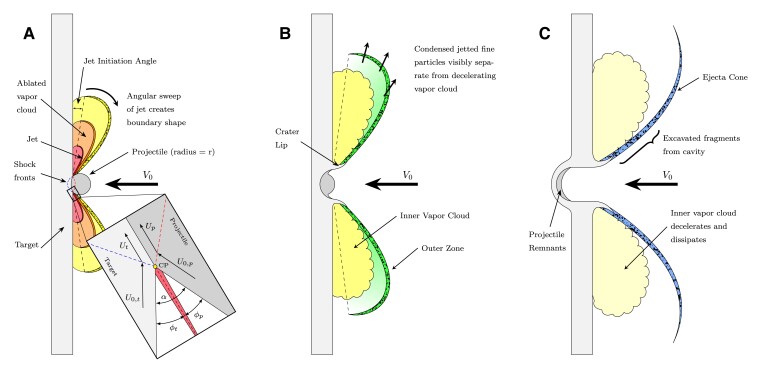
Cross sections of impact flash at early, transition, and late stages (not to scale). The initial stages of impact and flash formation by means of impact jetting are illustrated in A). The characteristic time, $\tau =r_{i}/V_{0}$, represents the limiting timescale over which jet formation can occur. After initiation and during initial penetration, the impact jet sweeps out from the target creating an expanding annulus of radiating material. The collision point centered (CP) frame of reference used in jet initiation calculations is illustrated. The post-jetting ejection behavior begins to transition B) to excavation of the impact crater. As the vapor cloud expands, cools, and decelerates relative to jetted particles, condensed phase material visibly separates from the vapor cloud. At later times, cavity excavation dominates the generation of fragments and the familiar ejecta cone develops fully C). The target is nearly fully perforated, as shown. The radiating vapor cloud dims and begins to dissipate.

Multiple mechanisms may contribute to a given impact flash (11), but for these impact velocities (3 km s${}^{-1}$ at normal incidence) we interpret our observations within the framework shown in Fig. 3. The impact event itself is divided into two primary stages: early-stage impact (during which the impact flash dominates observations) and late-stage impact and ejection (during which the impact crater develops and target perforation occurs). The representative timescale $\tau =r_{i}/V_{0}$ (where $r_{i}$ is the projectile radius and $V_{0}$ the impact velocity) separates early-stage times from late-stage times. During first contact at these velocities, an impact jet forms due to the acute wedge angle (α) formed by the colliding surfaces of the target and impactor (Fig. 3A inset). (30) This jet accelerates highly shocked and heated material outwards from the collision point of impactor and target. As the spherical projectile penetrates, the collision point and vertex of the wedge (wedge angle α) move outward, and the impact jet sweeps away from the target plane, such that material is jetted at steeper angles relative to the target surface. As the jet sweeps away from the target, the velocity of the jetted material also decreases (34). The trajectories of jetted material and the nature of this sweep determines the morphology of the phenomena observed at early times. In general, the extreme shock pressures generating the jet may be intense enough to melt and vaporize material. However, impactor speeds in excess of those investigated here are required to directly shock vaporize the materials used here, as illustrated by Zhaoxia et al. (9, 11). As such, we know of no impact mechanism energetic enough to vaporize substantial material that would be active under these impact conditions. We therefore hypothesize, in agreement with previous works (11, 14), that the observed vapor-phase emission must result from the vaporization of condensed-phase material caused by the interaction of impact ejecta with the surrounding ambient atmosphere rather than by impact heating itself.

As the jetted material forming the flash expands, the apparent vapor cloud—which we attribute to the ablation of very small condensed particles, similar to that suggested by Sugita and Schultz (14)—decelerates and dims. Radiating condensed-phase particles and fragments penetrate and separate from the observable boundary of the vapor cloud, creating the particular visual structure observed in Fig. 2D. As the projectile continues to penetrate the target, jetting terminates and a crater forms; the large-scale ejection of solid fragments by the excavation of this crater forms the dark, visible front-surface ejecta cone traditionally associated with penetrating hypervelocity impacts (Fig. 3B). While we cannot perform experiments in hard vacuum, this mechanistic interpretation suggests that if these specific impacts occurred in ultra-high vacuum there would be negligible impact flash, the remaining radiation likely being continuum in nature, attributable to the greybody surface radiation of hot condensed-phase ejecta particles. Such condensed-phase surface radiation likely contributes in part to lunar impact flash occurring in ultra-high vacuum (27).

**Fig. 4.**
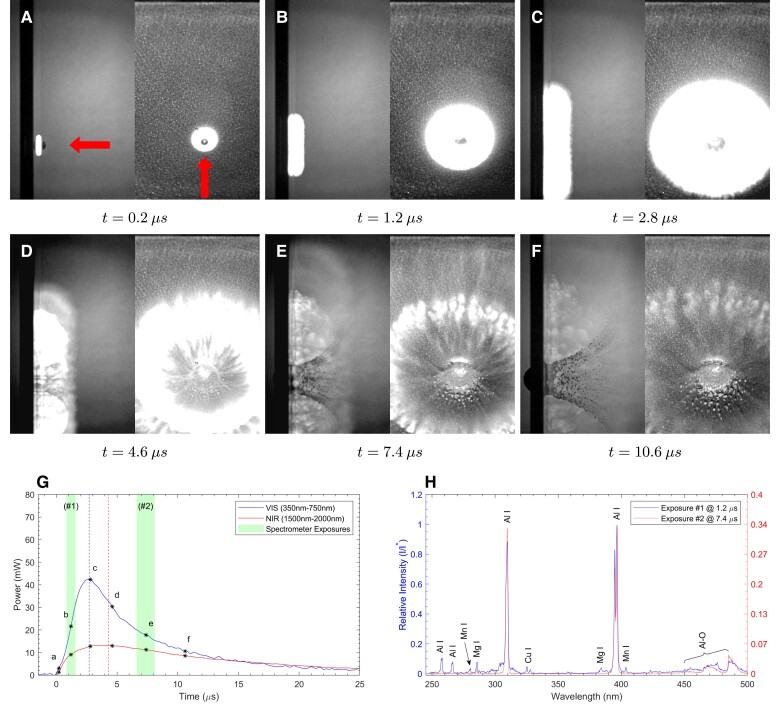
2024Al projectile impacting 6061Al target at 90${}^{\circ }$ obliquity with normal velocity of 3.2 km s${}^{-1}$ in 10 Torr air. See Fig. 2 for description of figure elements.

**Fig. 5.**
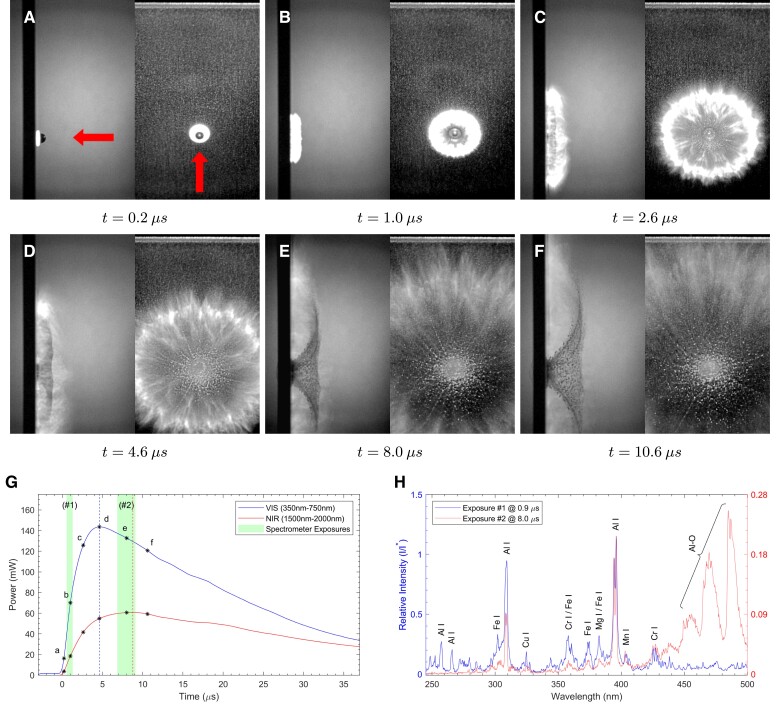
2024-Al Projectile Impacting 304SS Target at 90${}^{\circ }$ obliquity with normal Velocity of 3.1 km s${}^{-1}$ in 15 Torr Air. See Fig. 2 for description of figure elements.

The operative mechanisms can be better understood by performing similar experiments using reciprocal and symmetric combinations of target and projectile materials, since the signatures of the mechanisms are strongly affected by the materials themselves. In addition to stainless steel impacting aluminum (SS/Al), we provide results for the symmetric case of Al impacting Al (Al/Al) and the reciprocal case of Al impacting SS (Al/SS), all at similar impact velocities. Obtaining the full set of diagnostics for each material pair allows identification and comparison of the key mechanisms in each case. We present the corresponding visualizations, photometry, and high-speed spectroscopy for these material pairs under normal incidence impact in Figs. 4 and 5. Relevant mechanical properties for all materials are presented in the Supplementary Table S1, together with results from additional oblique configurations in Supplementary Figs. S4–S6.

The symmetric materials case is shown in Fig. 4, with a 2024Al projectile impacting a 6061Al target at 3.2 km s${}^{-1}$. The high-speed optical images (Fig. 4A to F) show that the impact flash develops by a similar process, with an outer component whose boundary transitions into the classical ejecta cone, and expands faster radially along the target plate. However, the outer component is less distinct in this case. The evolution of the luminosity (Fig. 4G) differs from that in Fig. 2G, with the maximum in the NIR luminosity now occurring *after* the maximum in the optical luminosity. While Al peaks still dominate the atomic emission, the molecular behavior differs greatly in that no significant increase in Al-O emission relative to the Al atomic peaks is observed for this particular shot. The spectral shapes of both early and late exposures are generally more similar than was the case with the previous SS/Al impact shown in Fig. 2, and the overall peak intensities do not decay as much. Neither Figs. 2G nor 4G show prompt emission—meaning narrow, fast-rising luminosity spikes produced immediately after impact. Prompt emission (8–10) indicates the presence of material vaporized *directly* by impact processes. The absence of such spikes within the first microsecond after impact would appear to confirm that there is no significant impact-induced vaporization; however, the higher ambient pressure atmospheres used in this work (in excess of 1 Torr) may prevent clear observation of prompt emission spikes (9, 11). Impact experiments on metals performed by Zhaoxia (9) under higher vacuum, in which prompt spikes are observed above impact speeds above $5\;km\;s^{-1}$, also found no prompt emission at our impact speeds of 3 km s${}^{-1}$. Our observed vapor phase characteristic emission is thus attributed to the aerothermal ablation of fragments of condensed phase material that have broken off an impact jet.

To investigate the effect of atmospheric composition on flash emission and confirm combustion of Al with ambient oxygen, we performed a repeated Al/Al impact in a 10.6 Torr argon atmosphere. No Al-O molecular emission was observed despite a large increase in the overall intensity of the metallic peaks; this increase in flash intensity is consistent with an ablative mechanism as the reduced heat capacity of the monatomic argon would produce higher temperatures in the post-shock gas layer driving the ablation of a high-velocity fragment. Unfortunately, there are no particularly strong, easily observed emission lines of argon or air ($N,O,N_{2},O_{2},$ etc) within the measured spectral range. What potential atomic, ionic, or molecular lines might be present are expected to be heavily overpowered by the emission of even trace metallic species under equilibrium conditions (33). Note that even alloying elements of the projectile and target metals are expected to produce higher peak intensities than ambient gas species within the measured spectral range. More details pertinent to the effect of the argon atmosphere are given in the Supplementary Section S5 and Supplementary Fig. S7.

Qualitatively different phenomena are observed for the reciprocal materials arrangement, when a 2024Al sphere impacts a 304SS target plate at 3.1 km s${}^{-1}$ (Fig. 5). In this case the overall impact flash power was much higher, so that a 640 nm CWL-40 nm FWHM bandpass interference filter had to be used to allow proper visualization of the impact. As before, in Fig. 5A to C we see the initial radial and out-of-plane expansion of the bright emitting region. However, at later times, Fig. 5D to F, it is not clear that there is a separation between distinct inner and outer zones as brighter and darker emitting regions. The ejecta cone is also much shallower (Fig. 5E and F) than in previous configurations, and smaller ejecta particles are observed.

Fragmented particles are ejected from the developing crater as material flows radially out from the impact center and upwards from the target surface during target penetration. The flow of this material establishes the trajectories of late-stage ejecta fragments. Shallower craters provide shallower ejecta trajectories; as the crater deepens ejecta trajectories steepen relative to the target surface, usually approaching a steady, terminal value often referred to as the ejecta cone angle. In the case of the SS/Al and Al/Al impacts, steeply-walled deep transient craters form, leading to full perforation of the target. For Al impacting SS, there is insufficient impact energy to perforate this stronger target, leaving a shallow crater with correspondingly shallower wall angles at the target surface. Generally, lower density projectiles (e.g. polymer, aluminum) impacting denser targets (e.g. iron, steels, copper) will generate shallower craters with depth-to-diameter ratios of less than 0.4, whereas projectiles denser than their targets generate ratios of 0.6 or greater (35–38). Increased relative target strength shows similar effects, generating shallower craters (39, 40). Terminal ejecta cone angles likewise become more shallow in these cases (41, 42), as observed here.

Unlike the previous configurations, the continuity of ejecta cone and outer emission zone boundaries observed in Figs. 2 and 4 is not observed here, suggesting a change in the mechanisms. The outer flash boundary initially outruns the ejecta cone in Fig. 5C and D. Comparing Fig. 5G with Figs. 2G and 4G, we see that both the VIS and NIR peak luminosities far exceed those observed in the previous impacts (i.e. this configuration produces a much brighter flash). Comparing the spectra in Fig. 5H with those in Figs. 2H and 4H, we see that the Al-O emission dominates the observed spectrum in exposure #2 (note that additional molecular peaks in this series exist (25) beyond the 500 nm range). Additional wide-band, long duration spectra were collected for selected experiments to confirm that the Al atomic and Al-O molecular emission does indeed provide most of the visual radiation. For a 100 μs integration time after impact, almost all the emitted light from 350–900 nm is provided by the characteristic emission and combustion of aluminum vapor; no continuum radiation nor other strongly emitting species were observed for either Al/Al or Al/SS impacts. Further detail, as well as the collected wide-band spectra are given in Supplementary Section S6 and Supplementary Fig. S8.

## A jet-based mechanism for the impact flash

Impact jetting of condensed material that subsequently interacts with the atmosphere to generate the flash is the only known mechanism consistent with all of these observations and the existing literature relevant to the given impact conditions. The impact of a spherical projectile on a planar target provides an axisymmetric configuration with a monotonically increasing local wedge angle (α) between projectile and target surface. Under the conditions described, the convergence of blunt-body projectile and planar target generate a fundamentally asymmetric configuration with respect to local closure geometry, even if projectile and target materials are identical, and the appropriate asymmetric jetting theory must be considered (11, 13, 43, 44). The jet calculations performed here follow the methods described by Sugita and Schultz (13), Kurosawa (45), and Zhaoxia et al. (11) as detailed in the Supplementary Section S1 and Supplementary Fig. S1. The \observed differences between Figs. 5 and 4 are a direct result of projectile and target material properties under these experimental conditions: classical asymmetric jetting theory predicts the initiation of the impact jet to occur in either the projectile or target depending upon shock properties and impact conditions. As such, we expect the flash behavior to be governed primarily by the hydrodynamic properties of the materials involved, while the main ejecta cone excavated during later-stage projectile penetration is sensitive to the strength properties and failure behavior of the materials.

**Fig. 6.**
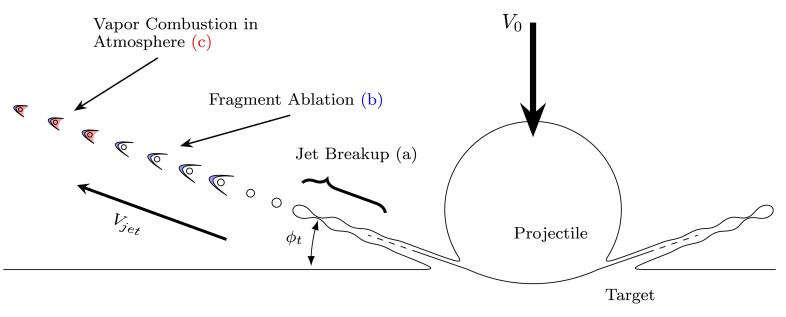
Lifetime of a jetted particle in the impact flash. The impact jet breaks up and fragments a), as a result of its generally axisymmetric expansion and radial velocity gradient effectively stretching and thinning the jet sheet. The interaction of the expanding jet with the atmosphere may also contribute to the breakup. It is likely that the jet is at least partially molten, so the proper mechanical description of this breakup is somewhat nebulous. The generated fragments, having a velocity $V_{\;jet}$ established by the deflection angle of the jet from the target plane, $\phi_{t}$, begin to ablate and decelerate due to their high-speed (approx. Mach 30) movement through the rarefied ambient atmosphere b). The ablated metallic vapor emits characteristically, producing identifiable atomic spectral peaks. As the ablated metallic vapor mixes with ambient oxygen, it combusts, producing the observed molecular Al-O emission. Note, the size of the jets and fragments in relation to the projectile are exaggerated here.

At the moment jetting begins, the impact jet resembles an expanding conical sheet originating from the annular contact locus of the impactor as it penetrates the target surface. During these early times, with t≪τ, the jet angle steepens and sweeps outward from the target as penetration continues (see Fig. 3A). Newly jetted material moves along steeper trajectories than previously jetted material, with generally reduced velocities as the shock pressures decay. Investigations of planar shock-induced jetting at free metallic surfaces have shown the breakup of the jet sheet begins at the jet tip and propagates backwards towards the base of the jet (46). We expect a similar breakup process to occur within a radially divergent metallic cone jet due to the development of instabilities (47), generating a distribution of fragment sizes (48). The impact jet is likely at least partially molten at these impact speeds. The jet breaks up as it expands radially as shown in Fig. 6, with the jetted fragments experiencing the sequence of events described in the figure. Jet fragmentation begins at a critical stretch for a given stretching rate (and therefore at a critical time). The jetted fragments ablate and decelerate as they travel at very high velocities (∼10 km s${}^{-1}$) through the rarefied chamber atmosphere. The smaller fragments will then aggresively ablate and decelerate, while the larger and more massive fragments continue to travel at high speed for longer. Micron or submicron fragments ablate quickly, producing an intense cloud of radiating vapor due to the large numbers of tiny fragments. As this vapor cloud decelerates and mixes with the ambient atmosphere, it combusts with residual oxygen generating the large amount of molecular emission that comes to dominate the flash at later times. The radiative intensity of an individual fragment is extremely sensitive to local relative slip velocity; quickly decelerated particles will decay in ablative intensity rapidly. The larger fragments continue to travel at high speed, and thus visibly separate from the vapor cloud as the overall flash intensity decays to observable values within the dynamic range of our imaging system. This process generates the two emission zones discussed previously.

**Fig. 7.**
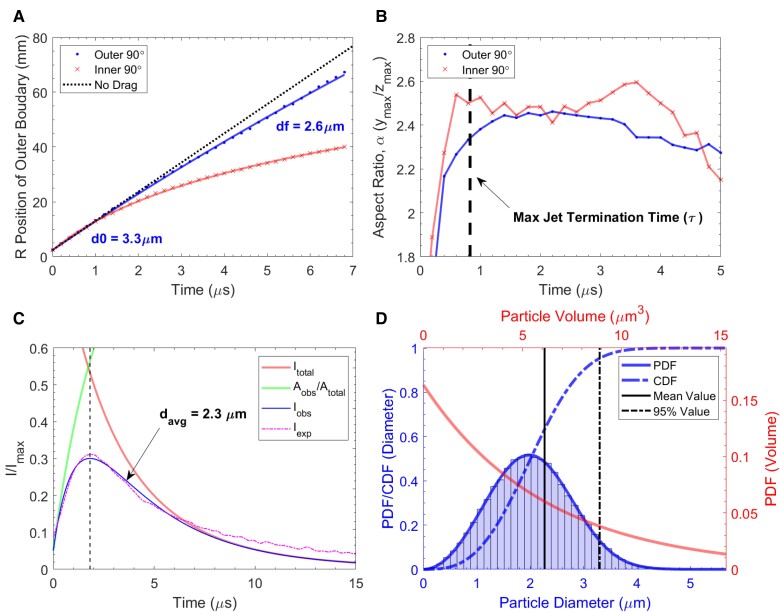
Al/Al flash expansion and luminosity analysis assuming particle cloud ablation. Experimental data presented for Al/Al impact at 3 km/s in 11 Torr air. The position of the outer emission front along the 10${}^{\circ }$ radial from the target plane A) was fit using a thermally ablative aerodynamic drag model assuming single sized spherical molten aluminum particles constituting the boundary of the flash. The aspect ratio of the flash given by the maximum (+*y* direction) height and (+*z* direction) width of the imaged cloud is given in B), and generally plateaus within τ, the maximum approximate limiting time over which jetting can occur. The observed variations in aspect ratio at times over 3 μs are due to degrading boundary definition in the experimental images as the flash intensity begins to decay. The calculated observable ablation luminosity ($I_{occ}$) of an expanding spherical shell particle cloud is given in C), assuming partial self-occlusion ratio $A_{obs}/A_{total}$. The calculated theoretical ablation intensity of the particle cloud is maximized here at t=0, where the initial velocity of each particle is highest. Zero time is shifted by $\tau =r_{i}/V_{0}$ to account for the formation time of the jetted particle cloud. Self-occlusion at early times diminishes the observable luminosity, and is assumed here to vary as the ratio of observable particle surface area ($A_{obs}$) to total particle area ($A_{total}$). The observable luminosity is fit to experimental data to determine an initial average fragment diameter. The particle distribution shape is assumed to be exponential in volume. The particle volume and size probability density distributions (PD) determined by the model fit shown in C) are given in D). The cumulative density (CD) of fragment size is also shown.

To measure the deceleration of the outer emission boundary, the edge of the flash region was tracked along a radial trajectory at an inclination of 10${}^{\circ }$ with respect to the target surface (this angle was chosen to correspond with the predicted critical jet initiation angle relative to the target surface). The motion of the outer emission boundary was fit with a thermally ablative drag model (49), previously applied to impact flash (11) but classically used for the analysis of meteor ablation. The results of the ablation model boundary fit are presented in Fig. 7A, with calculation details and model values given in the Supplementary Section S2 and Supplementary Table S2, respectively. Simulations (46, 50) and experimental measurements (51) of planar shock induced sheet jet breakup have suggested exponential distributions in fragment volume (such exponential distributions in fragment volume/mass are commonly applied to fragmentation problems (48)). The model fit suggests a mean initial particle diameter of approximately 3.3 μm for the ablating particles constituting the flash boundary.

We can also estimate the shape of the luminosity curve from the flash as a consequence of this jet-based mechanism. A thermally ablative drag model (9, 49) for spherical particles (see Supplementary Section S2) is used to estimate the luminosity generated by a particle size and velocity distribution in a prescribed ambient atmosphere. However, at early times some particles may block other radiating particles from sensor view, diminishing the observable luminosity of the flash cloud and imposing an effective “rise time” in the luminosity associated with particle cloud self-occlusion rather than ablative physics. Particle self-occlusion may therefore effectively control the rise behavior of the observed luminosity, and the peak luminosity may likewise be determined by the competition of the total decaying ablation luminosity of the cloud and the increasing visibility of that luminosity as self-occlusion decreases. The apparent rise time of the flash luminosity, at least in atmospheres dense enough to provide rapid ablation of fragments, may be a consequence of the interaction of these generative and occlusive terms. Further information regarding the effect of self-occlusion at early times in the particle cloud is included in the Supplementary Section S3 and Supplementary Fig. S3.

The experimentally observed luminosity of an Al/Al impact was fit assuming the thermally ablative radiation of an exponential distribution of spherical particles, accounting for self-occlusion. The resulting average particle diameter and model luminosity curve is shown in Fig. 7C. This luminosity fit suggests a mean initial fragment diameter of 2.3 μm. The corresponding particle distribution is shown in Fig. 7D, where the particle volume follows an exponential distribution. The 3.3 μm size independently estimated through fitting the boundary position (Fig. 7A) would represent approximately the 95th percentile fragment size in this assumed distribution. It is reasonable to expect the fit to the emission zone boundary motion to reflect a fraction of larger size particles that maintain their speed, and in this sense the particle size estimate from the luminosity calculation and the particle size estimate from the emission zone boundary measurement are consistent. While these approximate analytical calculations should be taken only as very rough assessments of fragment size assuming a common radiative mechanism, the reasonable agreement in estimated particle sizes accomplished by independent measurements—flash boundary position and visual luminosity—still supports the hypothesized ablation of particles as the source of the impact flash in this case. The shape of the model luminosity curve does not fully reproduce the decay of the experimental curve; a better description of the radiation physics involved is almost certainly necessary to improve this fit. The classical thermal meteor ablation model used here does not account for alternative erosive mechanisms that may be at play in the largely molten fragments or for the evolution of the radiating vapor, for example. One potential source of energy that may affect the later decay of overall observed VIS luminosity is the reaction of ablated vapor with atmospheric oxygen; oxide emission in the VIS range tends to dominate the emitted spectra at later times.

Note that the calculation of lab frame jet velocity in standard jetting theory uses a parameter *f* (30) that relates the jet velocity to the effective flow velocity of material into the collision point (indicated by point CP in Fig. 3A). Foundational work on jetting showed that f≈1 for colliding thin plates. (29, 30). Kurosawa’s experiments showed that such a theory overpredicted measured jet velocities by approximately 30% (45) using polycarbonate projectiles with similar impact geometry and velocities to this work. They found f≈0.4−0.8 depending upon impact conditions and materials. This discrepancy can be explained in part by the decay of shock pressure during spherical jetting as the newly initiated jet envelops and expels mass. It takes some time after initiation for substantial mass to be engulfed into the jet, and by this time the driving pressure associated with the jet may have decayed (34, 52). Here, we calculate an experimental value of *f* similarly to Kurosawa et al. (45) (see Supplementary Equation (5)). The resulting experimentally determined *f* values, ideal jet velocities, relevant experimental conditions, and experimental jet velocities (measured by ablative drag fit of the outer flash boundary positions shown in Supplementary Fig. S2) for all configurations tested are shown in Table 1. The standard theory (f=1 at critical initiation) overpredicts the jet velocities by 60–90%. Our experimentally estimated *f* values are much lower than those measured by Kurosawa. This large discrepancy may reflect our modeling approximations.

**Table 1.** Maximum experimental ($V_{\;jet,exp}$) and theoretical ($V_{\;jet,thr}$) jet velocities for all configurations.

	Al/Al		SS/Al		Al/SS	
	90${}^{\circ }$	45${}^{\circ }$	90${}^{\circ }$	45${}^{\circ }$	90${}^{\circ }$	45${}^{\circ }$
$P_{atmo}$ (Torr)	9.8	15.0	15.2	15.0	15.0	15.3
$V_{impact}$ (km/s)	3.21	3.11	3.02	3.03	3.07	3.03
$P_{\;jet,max}$ (GPa)	57.1	38.7	70.6	61.5	73.9	51.1
$V_{\;jet,exp}$ (km/s)	11.6	11.4	12.3	12.0	11.3	11.5
$V_{\;jet,the}$ (km/s)	19.9	20.5	21.2	19.1	20.7	22.0
$f_{exp}$	0.17	0.09	0.16	0.21	0.10	≈0

The maximum hydrodynamic shock pressure achieved in the jet ($P_{\;jet,max}$) and the ideal jet velocity ($V_{\;jet,thr}$) are calculated assuming critical initiation conditions. Real experimental projectile impact speeds and ambient pressures are also given.

## Jet initiation and the distinctive behavior of Al on SS impacts

**Fig. 8.**
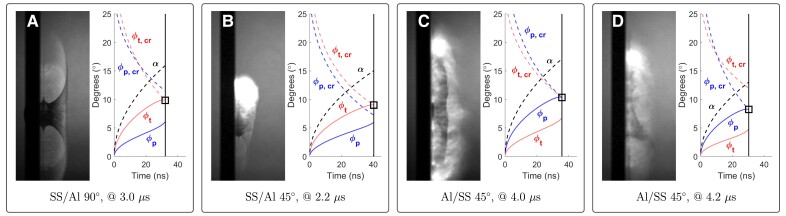
Flash/ejecta behavior and predicted jet initiation. Selected narrow-band filtered images from the SS/Al and Al/SS impacts are shown. The full experimental results for B) and D) are given in Supplementary Figs. S4 and S6, respectively. The deflection angle of the material flow in the collisional frame is given by ϕ. Jet initiation is predicted when ϕ reaches its respective critical value, $\phi_{cr}$, such that $\phi_{cr}-\phi =0$. The initiation conditions are boxed in the figure. In the case of the SS/Al configurations, jet initiation is predicted to occur in the target A,B). However, jet initiation is predicted to occur in the projectile for the Al/SS configurations C,D). The wedge angle between projectile and target surfaces is given by α, and the initiation time is marked by the vertical line.

Our impacts are fundamentally asymmetric with respect to jet initiation. Thus one side of the impact will reach criticality—when the deflection angle, ϕ, associated with the oblique shock in either target or projectile exceeds the critical angle, $\phi_{cr}$, as indicated in Fig. 3A—initiating the jet in the critical material (see Supplementary Section S1 and Supplementary Fig. S1). While it is expected and observed that material on the non-initiating side is quickly entrained into the jet, the predicted initiation of the jet still provides insight into the nature of early-time material ejection during an impact. Our predicted jet initiations for the SS/Al and Al/SS impact configurations are shown in Fig. 8. The jet is predicted to initiate in the target material for the SS/Al configurations, but in the projectile material for the Al/SS configurations. Indeed, the materials used here have sufficiently different wave speeds that the Al structure will see critical deflection first in all cases. This gives us potential insight into why there is a change in flash/ejecta boundary continuity when the projectile and target materials are swapped.

When SS impacts Al, the jet is predicted to initiate in the target (Fig. 8A and B). Note that the jet evolution and associated flash behavior also depend upon the hydrodynamic properties of the non-initiating side (the projectile in this case). As the jet terminates, the material ejection mechanism transitions to excavation of the impact crater. The excavation process under these impact conditions depends upon the interaction of both projectile and target during penetration, but the trajectories of excavated ejecta are strongly linked to the evolution of the crater geometry (controlled by the target material response). In this configuration, the ejected fragment trajectories are primarily controlled by the target side material at both stages and the transition from early-time jetting to late-time excavation involves processes dominated by the same material characteristics, that of the target. This smooth mechanistic transition provides a continuous set of fragment trajectories as jetting subsides and crater ejection begins. This manifests in the observed continuous transition of flash boundary to ejecta cone boundary in SS/Al impacts.

In the case of both normal and oblique Al on SS impacts, the jet is predicted to initiate on the projectile side (Fig. 8C and D), as opposed to the target side. As such, the material side controlling the trajectories of ejected fragments may effectively switch from projectile to target as early-stage jetting transitions to later-stage crater excavation. This jump from projectile-controlled ejection to target-controlled ejection may result in the observed lack of continuity between the flash and ejecta cone boundaries when Al impacts SS. In contrast to the previous case, jetting and excavation must be considered as distinct and separate mechanisms controlled by different material properties (the projectile during jetting and mostly the target during excavation). Note, eroded projectile material comprises much of the solid excavated ejecta in the Al/SS configuration, as the crater development and size is limited, but the crater geometry and shape are largely governed by the steel target material properties and therefore the character of the excavation process is still heavily target dependent. However, more work is needed to understand and quantify these mechanisms.

The contrast between continuous and discontinuous jet and ejection behavior could provide a strong signature for future models capable of capturing both multi-phase impact jetting and crater growth phenomena. The traditional limitations of the hydrocodes often used to model shock physics and Lagrangian finite element methods commonly used to model solid mechanics phenomena have usually required researchers to target specific mechanisms, and even cutting edge modeling methods are challenged by multiple physical states and mechanisms. However, to advance physical understanding of such complex coupled phenomena as the effects of impact-generated ablative fragment clouds on the aerodynamics of surfaces and supersonic boundary flows, more detailed experiments addressing mechanisms across the full range of the problem will be required. Future multiphysics modeling approaches will need a variety of empirical signatures for validation, and phenomena similar to those observed in these experiments may provide just those valuable signatures.

## Conclusion

Previous hypervelocity impact studies have separately investigated the phenomena of impact flash and the mechanics of material ejection in various impact configurations. The experiments performed here interrogate both flash and ejecta in one system by using in situ time-resolved diagnostics, and we have observed several interesting connections between these two phenomena in metals. Our experiments demonstrate a mechanistic connection between radiating flash phenomena and ejecta cone formation, a phenomenon linking several ejecta producing mechanisms across impact relevant timescales. We show that under these impact conditions the impact flash is seemingly driven by ablation of particles fragmented from the impact jet, and that the interaction between ambient atmosphere and high-speed condensed phase ejecta are the source of radiating vapor generating these surprisingly intense impact flashes. Despite lacking the energy necessary to impact vaporize material, by jetting or otherwise, the presence of substantial ambient atmosphere provides a mechanism of vaporization sufficient to produce intense flash emission. As such, these laboratory impact flashes belong to a separate mechanistic class than those produced by much higher velocity space environment impacts. A mechanistic viewpoint satisfying those phenomena would likewise fail to properly interpret flash phenomena such as particle impacts on high-velocity vehicles or perhaps even certain planetary impacts in rarefied atmospheres such as Mars. Furthermore, this work suggests we may be able to inform analysis of jetted particle size based upon observations of the impact flash evolution alone. Supported by refined radiative models, this approach may provide a way to interrogate highly dynamic condensed matter impact and jetting phenomena at larger spatio-temporal measurement scales.

## Experimental methods

**Fig. 9.**
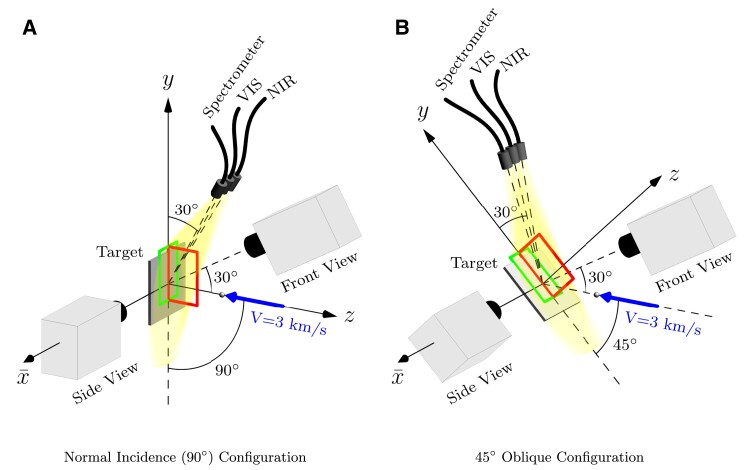
Impact and diagnostic configurations. Both the side view camera field-of-view and front camera field-of-view are approximately 65mm(w)×105mm(h). The side view camera is rotated with the target for oblique impacts, such that the coordinate axes are maintained with respect to the target surface. The front view is fixed with respect to the projectile velocity vector. Spectrometer, VIS, and, NIR luminosity collection optics are oriented at 30${}^{\circ }$ to the target plane, collecting roughly an $8^{\prime \prime }$ diameter field-of-view at the target and fiber coupled to the UV/VIS spectrometer and photodiodes, respectively. The collection optics view the impact from the top of the impact chamber to reduce coupling of camera illumination to the VIS detector and are rotated with the target to maintain relative orientation to the target surface under oblique impact.

All of the experiments were performed at the Hopkins Extreme Material Institute HyFIRE (Hypervelocity Facility for Impact Research Experiments) facility. The facility was designed to provide experimental versatility over a variety of high-velocity and hypervelocity impact conditions, and provides flexible *in situ* time-resolved diagnostics (53) with an emphasis on quantification and visualization. The core launch capability in HyFIRE is a small-bore (7.62 mm) two-stage light gas gun that provides launch velocities exceeding 5 km s${}^{-1}$ for 1 g launch packages. Our experiments studied the mechanisms developed during the high-velocity impact (at 3 km s${}^{-1}$) of 5 mm spheres onto 6.35 mm thick target plates at normal incidence (90${}^{\circ }$) and 45${}^{\circ }$ impact obliquity. The impactors were made of either 2024 aluminum alloy (2024 Al) or 304 stainless steel (304SS), while the target plates were made of either 6061-T6 aluminum (6061 Al) or 304SS. Thus the projectile/target material combinations were 2024Al/6061Al (Al/Al), 304SS/6061Al (SS/Al), and 2024Al/304SS (Al/SS). All of the impacts occurred in air within a target chamber evacuated to approximately 10–15 Torr. The target and diagnostic configurations are shown in Fig. 9; the diagnostics used consisted of optical ultra-high-speed video, emission spectroscopy, and broadband luminosity monitoring.

Optical ultra-high-speed video was configured to observe both the optically radiating material contributing to the early impact flash (referred to here as “hot” ejected material) and the solid material and fragments forming the traditional late-stage ejecta cone (referred to as “cold” ejected material). Two Shimadzu HPV-X2 cameras were used to view the impact, one oriented to view the impact perpendicular to the shot axis (Side View in Fig. 9) with the target in profile, and the second oriented to view the target front (impacted) surface from an angle 30${}^{\circ }$ from the shot axis (Front View in Fig. 9, and thus orthogonal to the lens axis of the first camera). Illumination was provided by a synchronized Cavitar illumination laser providing reflected illumination for the front camera, and diffused monochromatic backlighting for the side view camera (54). All data presented were captured at 5 Mfps with approximately 110 ns exposures. The backlight illumination was configured to provide center region mid-level grayscale, darkening towards the periphery of the frame, so that hot expanding material and cold center ejecta could be tracked with high contrast in any single experiment. Monochromatic illumination was used to facilitate partial bandpass filtering of the flash when desired, without affecting the cold material contrast; this also allowed simultaneous emission spectroscopy and impact flash monitoring without interference from broadband illumination sources. For all Al/SS impacts, the high impact flash intensity demanded the use of 650 nm CWL imaging intereference filters with 40 nm FWHM passbands to partially suppress flash intensity.

Optical impact flash emission spectra were collected using a Princeton Instruments SCT-320 imaging spectrometer coupled to a PIMAX4 1024i (200–900 nm spectral range, with 1024×1024 pixel detector) interline ICCD camera that allows the collection of multiple gated, intensified exposures over the duration of a typical event. Two UV/VIS spectrometer exposures were taken during each impact experiment; the first exposure triggered as soon as possible after impact (typically centered about 1 μs after impact), and the second exposure was centered approximately 8 μs after impact. The duration of and specific timing of each exposure is noted in the results section, but the first exposure was typically 750 ns long (occurring before peak flash visual intensity) and the second exposure was typically three times as long to help balance signal intensity with the brighter early exposure. Deuterium and halogen broadband sources were used to correct the relative spectrometer response across the UV-VIS spectral range. The spectrometer collection itself was triggered by the rising signal from an amplified Si photodiode (VIS detector in Fig. 9) collecting visual emission from the impact site. An amplified InGAs photodiode collected near-infrared emission (NIR detector) with similar optics and viewing as accompanied the visual detector. The photodiode optics were filtered such that the VIS detector covered a spectral range from 300–750 nm, while the NIR detector covered 1500–2000 nm. A similarly mounted, polarization-resistant fiber-coupled UV fused silica optic with approximately the same FOV as the photodiodes provided UV/VIS emission to the spectrometer. The emission spectrum from approximately 250–500 nm is analyzed in the present work, requiring the use of UV silica window insets on the top chamber bulkhead to transmit the required wavelengths. The resulting configuration provided in situ time-resolved luminous intensity measurements over the 300–750 nm VIS and 1500–2000 nm NIR bands, as well as gated emission spectra from 250–500 nm collected from the same FOV and viewing angle.

## Supplementary Material

pgad214_Supplementary_Data

## Data Availability

All data pertaining to the current study are publicly available at Craedl.org. https://doi.org/10.57695/kq0x-m552.
