# Supplementary material for: First contact: Fine structure of the impact flash and ejecta during hypervelocity impact
Source: PNAS Nexus. 2023 Jul 11;2(7):pgad214. doi: 10.1093/pnasnexus/pgad214 (PMC10335349; doi:10.1093/pnasnexus/pgad214)
Supplement: pgad214_Supplementary_Data [file pgad214_supplementary_data.zip › PNASNEXUS-PNASNEXUS-2023-00135R-s03.pdf]

PNAS nexus

Supplementary Information for

First Contact: Fine Structure of the Impact Flash and Ejecta during Hypervelocity Impact

Gary Simpson[1,2], Justin Moreno[2], Matthew Shaeffer[2], and K.T. Ramesh[1,2]

[1]Mechanical Engineering, Johns Hopkins University, Baltimore, 21218, MD, USA
[2]Hopkins Extreme Materials Institute, Johns Hopkins University, 21218, MD, USA

Corresponding Author: KT Ramesh
Email: ramesh@jhu.edu

This PDF file includes:

Supplementary text sections S1 to S6
Figures S1 to S8
Tables S1 to S2
Supplementary References

S1 Jet Initiation Calculations

A more detailed description of standard theory jetting calculations is given by Kurosawa[1], Vickery[2], Sugita and Schultz[3] and Zhaoxia[4] but a short summary of the procedure used in this work is given here. The geometry used in this analysis is reproduced in Supplementary Fig. S1. The wedge angle subtended by the target and projectile surface during penetration, α, is determined by (1a) where V_0 is the impact velocity, θ is the impact obliquity angle (normal incidence is θ=90°), r_i is the projectile radius, and t is the time after initial impact. In an impact between different materials—the asymmetric case—the deflection angles for the projectile and target sides, ϕ_p and ϕ_t respectively in (1b), sum to the wedge angle.

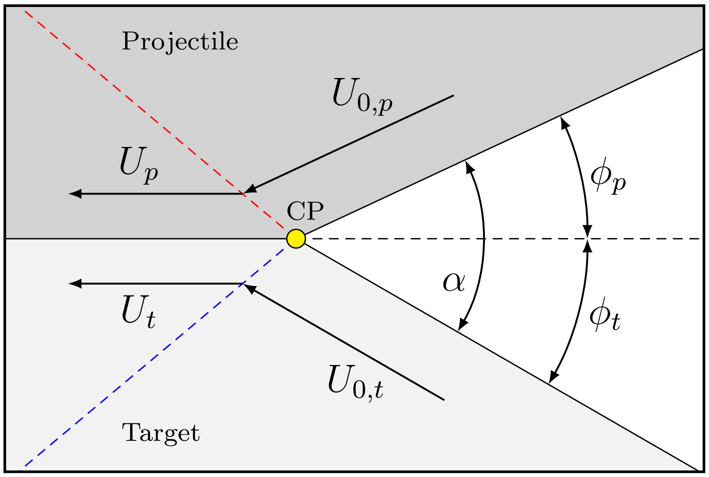

Supplementary Fig. S1: The collision point (CP) frame of reference used in standard jet theory calculations. The effective impact velocities in the projectile and target, $U_{0,p}$ and $U_{0,t}$ respectively give the velocities of material flow into the oblique shocks attached to the collision point (CP). The oblique shocks deflect the material flow along the slip-line interface between projectile and target. The sum of the projectile and target flow deflection angles,ϕ_p and ϕ_t, give the wedge angle subtended by the projectile and target free surfaces, α. The jet is predicted to initiate in the material whose deflection angles meets it critical value first.

$$\alpha = \arccos\left(1 - \frac{V_0\, sin(\theta)\, t}{r_i}\right) \tag{1a}$$

$$\alpha = \phi_p\,(U_{0,\,p}) + \phi_t\,(U_{0,\,t}) \tag{1b}$$

Within the frame of reference centered on the collision point, CP, where the penetration projectile surface meets the target surface, the effective impact velocity U_0 in the projectile and target are given by (2a) and (2b), respectively. The effective velocity represents the flow velocity of unshocked material into the oblique shock fronts attached to the collision points.

$$U_{0,\,p} = \frac{V_0\, sin(\theta)}{sin(\alpha)} \tag{2a}$$

$$U_{0,\,t} = \frac{V_0\, sin(\theta)}{tan(\alpha)} + V_0\, cos(\theta) \tag{2b}$$

Table S1: Mechanical Properties For all Projectile and Target Materials.[5,6]

	2024 Al	6061 Al	304 SS
$\rho_0\,(kg/m^3)$	2784	2703	7890
$c_0\,(m/s)$	5370	5350	4560
s	1.29	1.34	1.49
$\sigma_y\,(MPa)$	345	276	215
$\sigma_{ult}\,(MPa)$	483	310	505

The relationship between projectile and target is established by the pressure continuity across the material interface, called the slip line. In this case, pressure in the shocked material must be continuous across the slip line (3c) but tangential velocity at the interface may be discontinuous such that material may mathematically slip along the interface. The pressure (3b) in each shocked material is therefore determined by the shocked compression, μ given in (3a), and the specific material properties. It should be noted that a typical linear $U_s - U_p$ relation is used for all projectile and target materials here, where c_0 is the bulk sound speed and s is the slope term coefficient. The values of all material properties are given in Table S1.

$$\mu = \frac{\rho_s}{\rho_0} - 1 \tag{3a}$$

$$P = \frac{\rho\, c_0^2\, \mu(\mu+1)}{(\mu(1-s)+1)^2} \tag{3b}$$

$$P\,(\mu_p) = P\,(\mu_t) \tag{3c}$$

The deflection angle associated with an oblique shock achieving a given compression, μ, is given by (4a). The calculation of the deflection angles for a given wedge angle, α, is accomplished by iterating over a pair of projectile and target deflection angles to find the combination of angles that satisfy the pressure continuity required by (3c).

$$tan^2(\phi) = c_0^2\mu^2 \frac{U_0^2\,(\mu(s-1)-1)^2 - c_0^2(\mu+1)^2}{[\,c_0^2\,\mu(\mu+1) - U_0^2\,(\mu(s-1)-1)^2\,]^{\,2}} \tag{4a}$$

$$\frac{d}{d\mu}\, tan^2(\phi)\Bigg|_{\mu=\mu_{cr}} = 0 \tag{4b}$$

As previously discussed, the initiation of a jet is predicted to occur when one material reaches it critical deflection angle, or alternately its critical compression, beyond which the associated oblique shock detaches from the collision point, CP, and moves upstream. Shocked material releases into the traction free wedge angle, creating a highly energetic jet of material. The critical deflection angle for a given material experiencing a effective velocity, U_0, can be determined by finding the maximum of (4a). Standard jetting theory therefore postulates that an impact jet initiates when a critical condition is met and $\Delta\phi = \phi_{cr} - \phi = 0$. The value of f, as introduced by Walsh[7], jet velocity in the collisional frame to the effective impact velocity. A value of 1 suggests that the effective impact velocity magnitude of the unshocked material is recovered fully in the velocity magnitude of the jet. To derive the jet velocity in the lab frame, the motion of the collisional frame (CP) must be considered. For comparison to the values measured by Kurosawa et al[1], the experimental value

of f is calculated in (5), which considers the theoretical jet velocity magnitude as the average of the ideal projectile and target side jet spee evaluated at the time of first, critical initiation. This averaging is done because the real, observable jet will have mass contributions from both sides regardless of the theoretical source of initiation.

$$f_{exp} = \frac{2(V_{jet,exp} - U_{0,t})}{(U_{0,t} + U_{0,p})cos(\phi_t)} \tag{5}$$

S2 Thermally Ablative Drag Model

The ablative drag model used in the present work is of the form detailed by Bronshten[8] and implemented by Zhaoxia et al[4]. It represents the classical model of thermal ablation commonly applied to meteor ablation in planetary atmospheres, and thermalizes a fraction of the flow energy dissipated in the drag of the particle to vaporize material and reduce the particle mass.

The general equation of motion (6a), mass ablation (6b), and particle luminosity (6c) are given below, where the particle mass is m, the free-stream velocity is V, and the particle cross-sectional area to the flow is A_c. The drag coefficient (C_d), ambient atmosphere density (ρ_{atmo}), heat transfer coefficient (Λ), and heat of ablation (Q) are assumed to be constant. The heat transfer coefficient (Λ) is the fraction of the kinetic energy of the oncoming stream that contributes to ablation of particle mass, and the heat of ablation (Q) is taken as the energy per unit mass to raise the temperature and vaporize an initially molten particle, assumed to have been melted during the jetting process. The coefficient of radiative efficiency (γ_0) is assumed to be constant for all ablating particles, and is therefore incorporated only as a normalization constant for the particle luminosity, I.

$$m\,\frac{dV}{dt} = -\frac{1}{2}C_d\,\rho_{atmo}V^2A_c \tag{6a}$$

$$\frac{dm}{dt} = -\frac{1}{2}\frac{\Lambda}{Q}\rho_{atmo}V^3A_c \tag{6b}$$

$$\frac{I}{\gamma_0} = -\frac{1}{2}\frac{dm}{dt}V^2 \tag{6c}$$

The assumption of spherical particle shape leads to the self-similar velocity dependent solution (7a) for particle radius (r) assuming an initial radius (r_0) and initial velocity (V_0). The governing equations are rewritten assuming spherical particle shape with constant density (ρ_{frag}) in (7b) and (7c). The ablation parameter K heavily determines the ablation and drag behavior of the particle. The assumption of thermal ablation may not be appropriate for the mass loss under the experimental conditions discussed, but this basic ablative drag model offers a good starting point and is sufficient to demonstrate and evaluate the nature of of a particle based flash mechanism.

$$r(t) = r_0\,e^{-K(V_0^2 - V(t)^2)} \tag{7a}$$

$$\frac{dV}{dt} = -\frac{3}{8}\alpha_0V^2\,e^{-K(V^2 - V_0^2)} \tag{7b}$$

$$\frac{dm}{dt} = 4\pi r^2\rho_{frag}\frac{dr}{dt} \tag{7c}$$

$$K = \frac{1}{6}\frac{\Lambda}{Q\,C_d} \tag{7d}$$

$$\alpha_0 = \frac{C_d\,\rho_{atmo}}{r_0\,\rho_{frag}} \tag{7e}$$

Table S2: Thermal Ablation Model Parameters

ρ_{frag}	$2375\ kg/m^3$	Λ	0.9
Q	$13500\ (kJ/kg)$	C_d	2

* Only 2024Al projectiles impacting 6061Al targets were analyzed with the model, so the mixed material nature of the jet in other configurations was not considered.

The thermal ablative drag model was fit to the outer flash-boundary position (shown in Fig. S2) for all impact configurations. The initial velocity determined by the position-history fit is taken as the initial jet velocity of the particles. Note, the radial position of the flash boundary is measured along the (in-camera-plane) radial vector 10° inclined from the target plane. This inclination approximates the initiation angle of the impact jet, the trajectory along which the maximum jet velocity is expected to occur. The measured jet velocities for all configurations are presented in Table 1. While material configuration and obliquity do affect the driving shock pressure and therefore the jet velocity, the difference is not very significant at these impactor speeds.

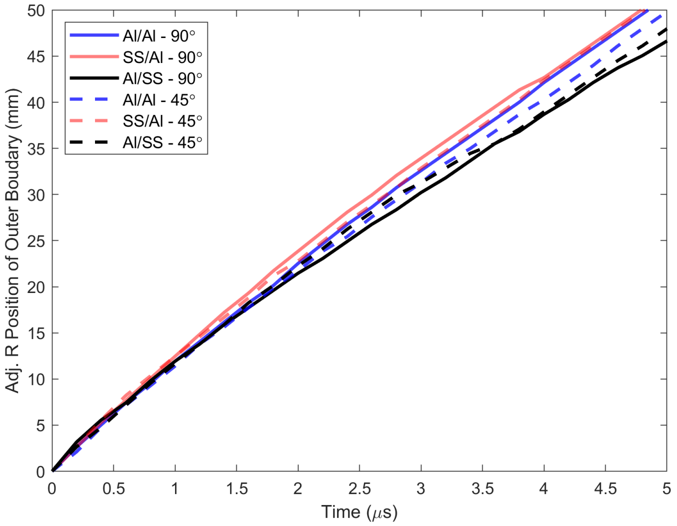

Supplementary Fig. S2: Measured emission front position and jet velocities (km/s) for All Experiments. The experimental jet velocities ($V_{jet,exp}$) measured by fitting the ablative drag model to the data presented here are given in Table 1.

S3 Particle Cloud Self Occlusion

The effect of particle self-occlusion must be considered under the assumption of a particle ablation driven impact flash. From the viewing perspective of a detector, a large fraction of the total particle radiant intensity may be blocked from view by other particles in the early, dense particle cloud. Here, we seek to estimate the effect of this occlusion by evaluating the fraction of particle surface area that is visible from a single perspective. To approximate a generalized particle cloud representing an impact flash we volumetrically expand a initial randomly packed sphere of particles following a prescribed size distribution. The number of particles particles participating in the impact flash is estimated assuming 5% of the projectile mass participating in the jet, based on prior work in the field. The very large number of particles implied by this jet mass requires a scaled calculation to be made. An exponential distribution in particle volume is assumed, and the calculation initial configuration is generated by the MATLAB based random-sphere-packing algorithms provided by Black and Cheviakov[9]. The resulting initial packed configuraiton of particles

is shown in Fig. S3A. The packed particle sphere is expanded volumetrically. The visible, non-overlapped fraction of particle surface area is calculated by Monte Carlo sampling of the projected particle surface areas projected on the y-z plane. Here, the view of the hypothetical detector is directed along the x-axis.

The calculation result for visible area fraction is shown in Fig. S3B. The shape is very well fit by exponential decaying towards full visibility. Some fraction of the initial area is of course visible to the detector at the start, given by f_0. This exponential shape is not unexpected. Grady[10] identified the applicability of Johnson-Mehl crystal growth dynamics to the problem of estimating fragment statistics experimentally in dense debris clouds. The random initial packing and volumetric velocity field resembles the growth of randomly nucleated circular crystals in the y-z plane, if the cloud is followed backward in time. Rather, starting at a large times where the particle cloud is diffuse and moving back in time, the particles appear to grow in relation to the cloud dimensions, eventually overlapping each other in the projected view. The overlapped area is analogous to the extended volume identified in the Johnson-Mehl model. The time behavior of the occlusion ratio, f_{occ}, is then characterized by the time constant, τ_{occ}.

The calculation must be scaled up to reflect the actual condition of the impact flash. The following scaling relations were developed to estimate the effective occlusion time constant, τ_{occ}, under experimental conditions by scaling a calculated, reference occlusion time constant fit in Fig. S3B, $\bar{\tau}$. Assuming an initial spherical mass of diameter D is divided into fragments of diameter d, the number of fragments, N, goes as (8b).

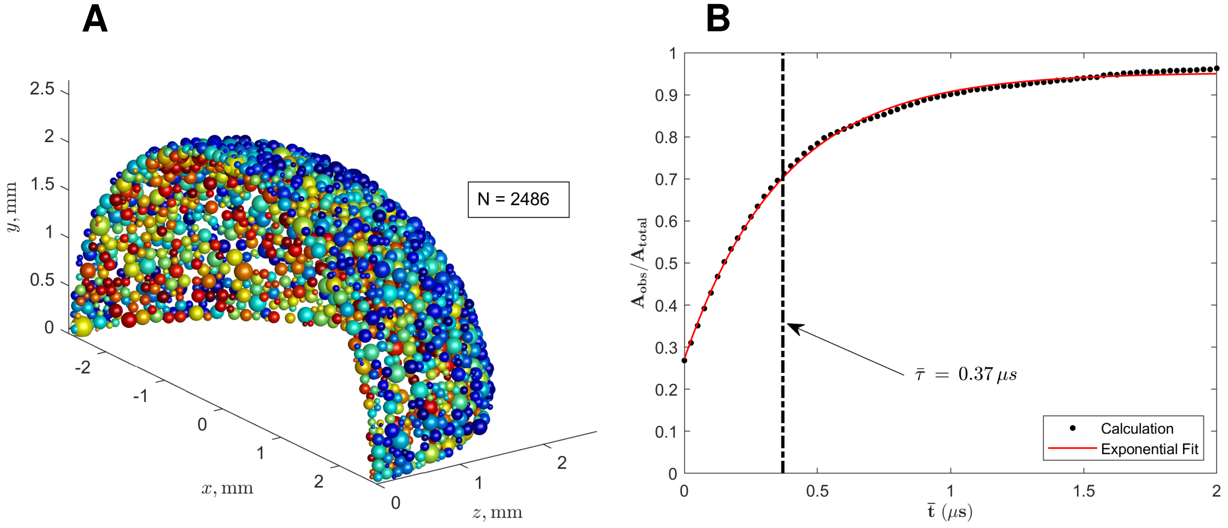

Scaled Calculation Particle Initial Configuration

Scaled Occlusion Area Ratio

Supplementary Fig. S3: The initial reference particle configuration consisting of randomly packed spheres is shown in (A). A linear radial velocity profile is applied to the particles in the shell, with the outermost particles traveling at the observed jet velocity of (10 km/s) and the innermost at the impact speed (3 km/s). The initial calculation configuration represents a shell of jetted particles originating at the projectile surface, distributed exponentially in particle volume with an average linear diameter of 110 μm, comprising a jetted mass of 5% the projectile total mass. The observable particle surface area viewable from the the detector (viewing along -x direction) is calculated via monte carlo sampling of the non-overlapping area projected on the Y-Z plane. The ratio of observable surface area to total projected particle area (sum total of circular particle area in Y-Z plane) for the expanded calculation cell (B) is fit using and exponential form (9a) and the resulting time constant is scaled according to (9c). The resulting occlusion ratio function (9a) roughly approximates the fraction of total radiant luminosity emitted from the surface of spherical particles that is visible to the detector in an expanding ablating particle cloud.

$$R_*^2 \propto N d^2 \tag{8a}$$

$$N \propto (\frac{D}{d})^3 \tag{8b}$$

$$t_* = R_*/V \simeq \frac{D}{V}\sqrt{\frac{D}{d}} \simeq \frac{D}{V} N^{1/6} \tag{8c}$$

If those fragments are expanded, forming our impact flash, such that no particles are self-occluded from a single perspective, then the minimum projected flash area accomplishing this goal will be the sum total of the particle projected areas. This minimum, no occlusion, cloud radius is identified as R_* in (8a). The timescale associated with this no occlusion condition is given by t_* (8c), where V is the expansion speed of the particle cloud boundary. This suggests that the timescale associated with full visibility for all the participating N fragments, for a given configuration and expansion velocity field, should scale roughly as the 1/6 power of the number of fragments, holding the expansion velocity of the cloud and the initial volume of participating mass constant.

$$f_{occ} = (1 - f_0)(1 - e^{(-t/\tau_{occ})}) + f_0 \tag{9a}$$

$$\tau_{occ} \propto \frac{R_0}{V} N^{1/6} \tag{9b}$$

$$\frac{\tau_{occ}}{\bar{\tau}} \simeq (\frac{R_0}{\bar{R}_0})(\frac{\bar{V}}{V})(\frac{N}{\bar{N}})^{1/6} \tag{9c}$$

As such, the occlusion time constant in the exponential form given by (9a) should scale as shown in (9b), and can be estimated for the experimental configuration using a calculated reference as shown in (9c). Note, the reference values apply to calculated configuration shown in Fig. S3A, with for example, $\bar{N} = 1659$. For the reference configuration shown, the initial packed configuration radius, $\bar{R}_0$, and expansion velocity magnitude $\bar{V}$, are chosen to reflect the experimental geometry and jet velocity magnitude such that the first two terms in (9c) are 1. The size distribution of fragments therefore determines N, where a large enough fragment size was chosen for the reference calculation to make $\bar{N}$ computationally practical. The experimental value of N is estimated using the experimentally determined fragment sizes discussed in the main body of the paper.

S4 Extension to Oblique Impacts

The mechanics of jetting, material ejection, and crater excavation all remain consistent in the case of oblique impact—where the projectile velocity vector is not normal to the target plane—but the general axisymmetry of the flash and ejecta cone is broken. General bilateral symmetry about the plane formed by the projectile impact velocity vector and the target plane normal is retained. We only consider 45° impact obliquity here; the 45° oblique configurations for both SS/Al and Al/Al are given in Supplementary Figs. S4 and S5, respectively.

In these configurations, the projectile has considerable momentum along the target plane surface. As a result, there is much more material excavated and ejected in the direction of the projectile's travel along the surface (+y direction) and there is corresponding asymmetry in the ejecta cone. This momentum also affects the jetting behavior and therefore the impact flash. The relative lateral motion of the projectile along the target plane affects the effective impact velocities $U_{0,p}$ and $U_{0,t}$ in the collision point frame of reference (given by supplementary equations (2a) and (2b) respectively) and therefore the jetting behavior of the system. Each point in the projectile contact locus (ring of contact points), experiences a different effective flow velocity and the jets developed there will initiate and evolve differently, but jets will form around the entirety of the contact locus. This behavior can be seen in the front view images of Figs. S4A-B

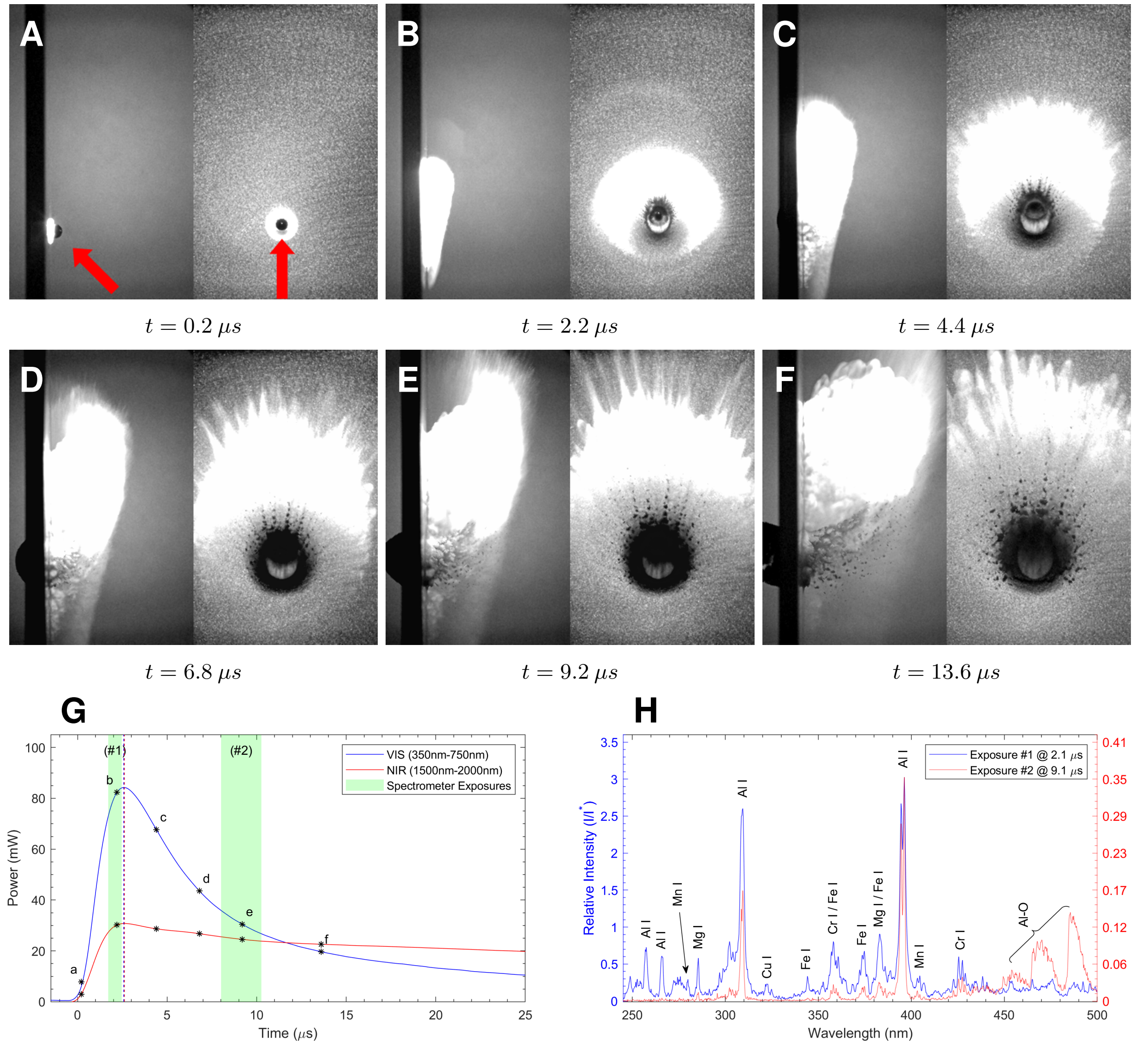

Supplementary Fig. S4: 304SS projectile impacting 6061Al target at 45° obliquity with axial velocity of 3.0 $km\,s^{-1}$ in 15 Torr air. See Fig. 2 for description of figure elements.

and S5A-B, as radiating material is initially jetted in all directions around the penetrating projectile. The inclined shape of the impact flash in side profile is determined by the altered set of trajectories achieved by this non-axisymmetric jetting behavior.

The concentration of high speed ejected particles in the direction of projectile motion leads to a very intense flash in the +y direction for the duration of the experiments and visible dark ejecta rays emanating from the crater in front view images at later times (D-F). The transit penetration of the ablated vapor cloud boundary by solid fragments is readily observable in these impacts, creating rays and bright streaks on the leading edge of the flash. The downrange concentration of fragment trajectories generates a intense asymmetric flash that ”travels” upwards in the field of view with the momentum vector of the projectile along the target surface. The radiative mechanism is fundamentally the same as the normal impact configuration. The impact obliquity does modify the distribution of fragment trajectories with subsequently affects the apparent flash structure as imaged. The peak VIS luminosity is largely similar between normal and oblique impacts, but the NIR peak luminosity is greatly increased under oblique conditions. In all cases, the total observed emitted VIS and NIR energy is much greater. Additional shear heating due to the projectile’s

momentum parallel to the target plane may possibly aid in the enhancement of the overall flash intensity by pre-heating ejected material. Additionally, the early time oblique emission spectra in Fig. S4 shows higher relative intensities of 304SS species peaks to 6061Al species peaks than the normal impact for the SS/Al configuration (Fig. 2). This may be an indication of higher relative participation of projectile material in the composition of the impact jet, as would be predicted at higher impact obliquity[3,11–13], but the variation in exposure timing between experiments makes direct comparison uncertain.

The 45° obliquity Al/SS impact (Fig. S6) displays somewhat bizarre behavior as the downrange edge (+y) of the shallow ejecta cone appears to extend back to the target surface before curving back away uprange (D-F). This distinct shape gives the visual impression of the ejecta somehow impacting the target surface and bouncing off; this is, of course, not accurate. Rather, the generally monotonic expected sweep of trajectories from shallow to steep seems to be interrupted, "skipping" back to shallow and slower solid fragment trajectories produced during the generation of the primary ejecta cone. The observed peak VIS luminosity in Fig. S6G is roughly 90% higher than that of the normal impact. NIR luminosity is substantially increased (G) over the corresponding normal impact, likely by a similar fraction as VIS, but in this single

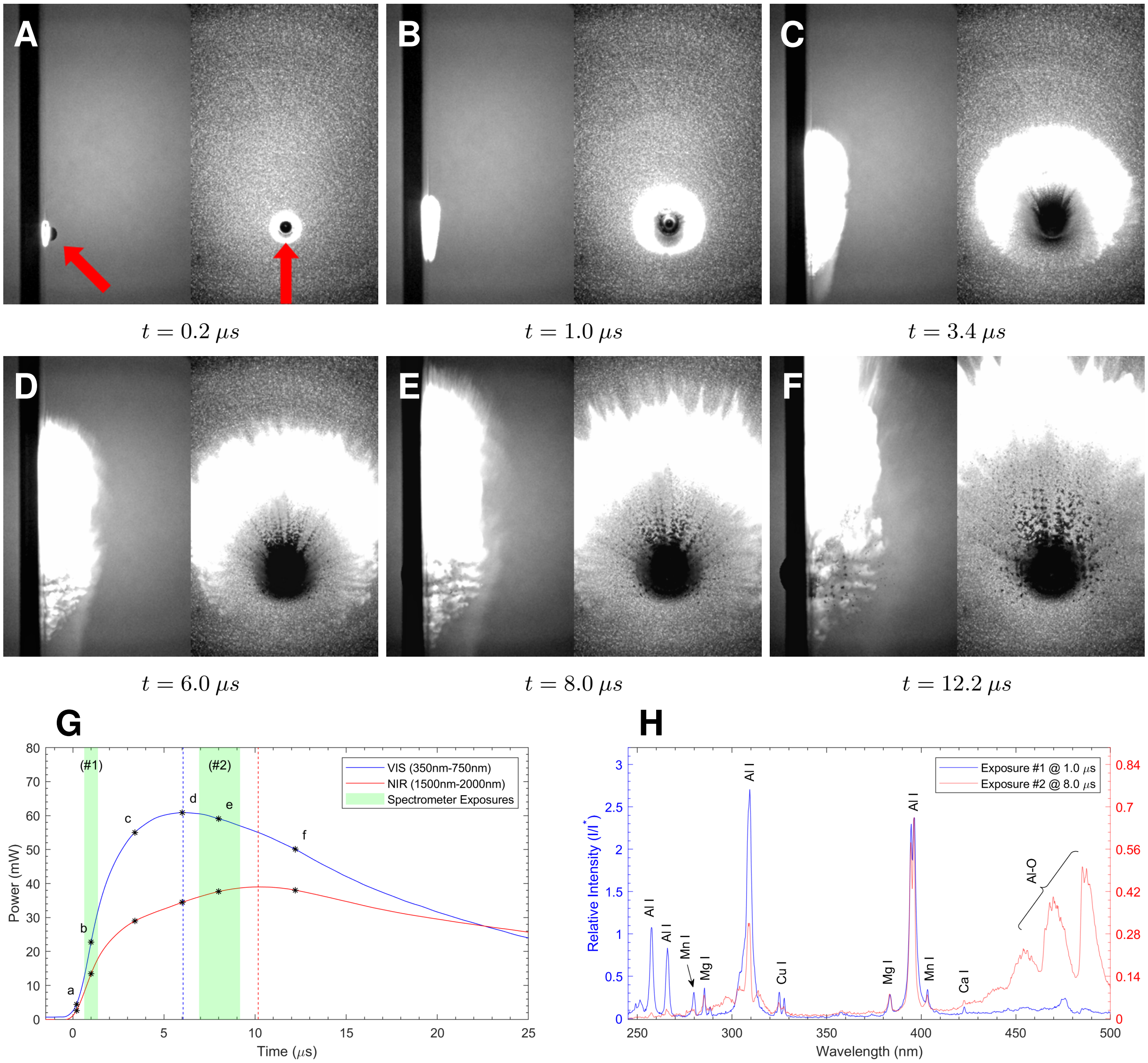

Supplementary Fig. S5: 2024Al projectile impacting 6061Al target at 45° obliquity with axial velocity of 3.1 $km\,s^{-1}$ in 15 Torr air. See Fig. 2 for description of figure elements.

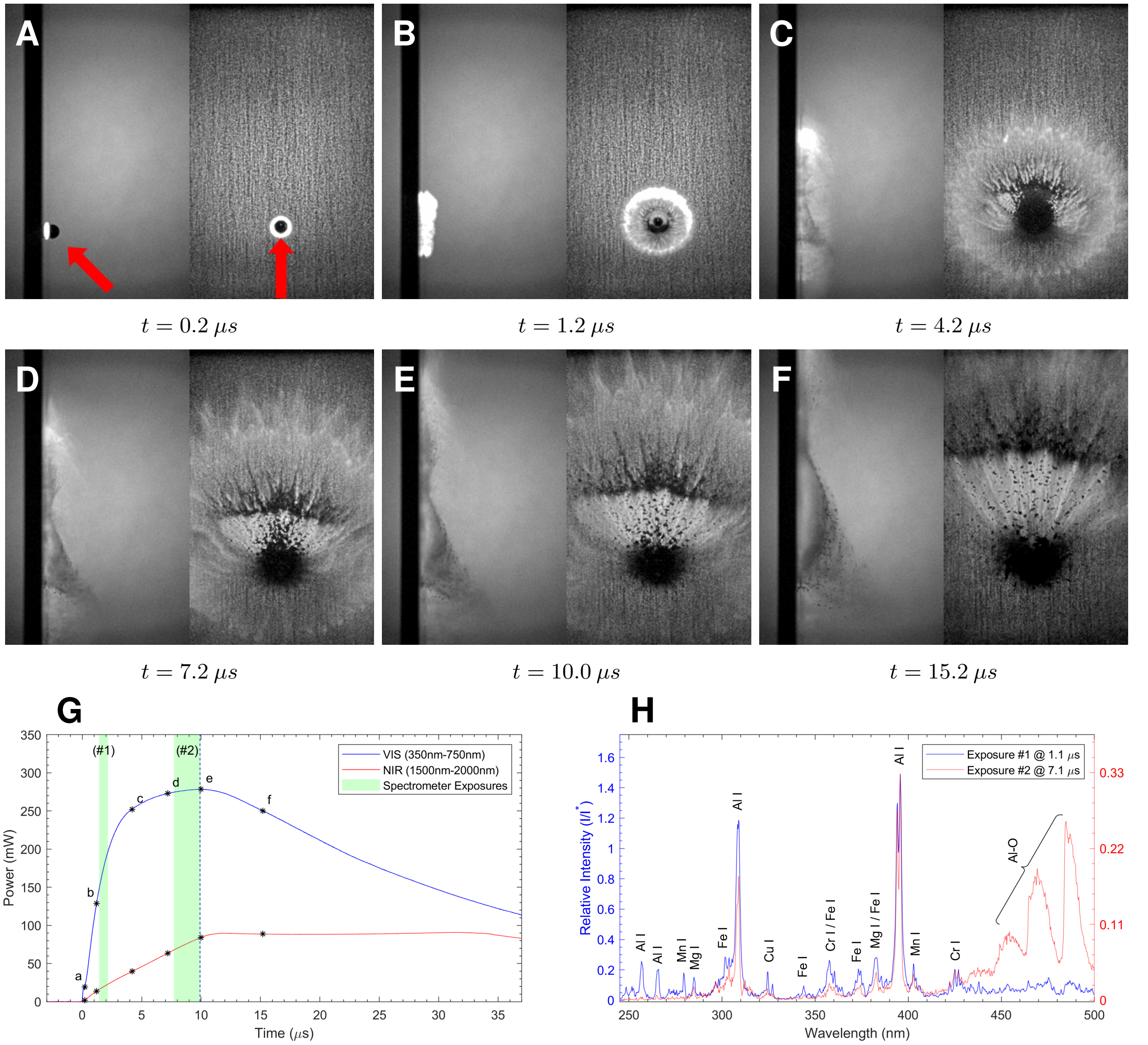

Supplementary Fig. S6: 2024-Al projectile impacting 304SS target at 45° obliquity with axial velocity of 3.0 $km\,s^{-1}$ in 15 Torr air. See Fig. 2 for description of figure elements.

case the detector saturated shortly after time (E) unfortunately making it difficult to assess magnitudes and NIR peak time. In both normal and oblique Al/SS impacts, the luminosity magnitude is so large that the decay is much slower than other configurations. Flash intensity in both bands persist long enough that issues with the observable extent of the expanding cloud, or even secondary impacts of high speed fragments in the test chamber may affect the data at longer times. However, similar to Fig. 5, the flash outruns the boundary of the the inclined ejecta cone in Fig. S6C, and the shape and extent of the flash zone bears little visual correlation with that of the solid ejecta cone. Once again, the smooth continuity between flash boundary and ejecta cone boundary that is observed for all SS/Al and Al/Al configurations is not seen when Al impacts SS. The smooth variation of ejected particle trajectories required to generate flash/ejecta boundary continuity implies some measure of continuity in the mechanisms generating ”hot” flash material and ”cold” ejecta fragments. The absence of boundary continuity in Al on SS impacts therefore implies discontinuous behavior between jetting and main ejecta formation mechanisms.

S5 Effects of Argon Atmosphere

To investigate the effect of ambient gas composition, including the aluminum combustion of ambient residual oxygen, the Al/Al impact configuration was repeated in a 10.6 Torr argon ambient atmosphere. Note, the pressure level used here is similar to that of the Al/Al impact conducted in 10 Torr air and the argon gas density is identical to that of the 15 Torr air atmosphere in which most experiments were conducted. The luminosity and emission spectra It is immediately apparent that the Al-O molecular emission peaks that tend to dominate the later stage flash emission have largely disappeared as expected in the inert atmosphere. The remaining emission may be due to some trace residual oxygen remaining in the impact range after argon backfill and evacuation, or may represent a contribution from multiple Ar II peaks. The strongest emission lines of argon —particularly atomic peaks—do not lie within the spectral measurement range used here, however, the overall intensities of the chief metallic flash contributions are much higher (Fig. S7B than those observed in ambient air of similar density and pressure.

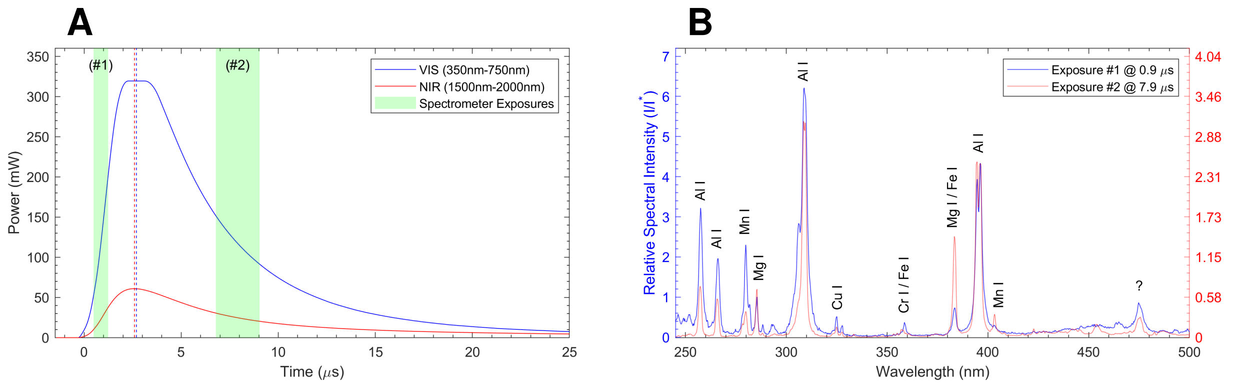

Supplementary Fig. S7: 2024Al projectile impacting 6061Al target at 90° obliquity with normal velocity of 3.1 $km\,s^{-1}$ in 10.6 Torr Argon. See Fig. 2 for description of figure elements.

The peak flash luminosity is seven times more intense in the visual band than the corresponding air impact (Fig. S7A). Argon emission may contribute to the time luminosity here as it is measured over a wider range of visual wavelengths (350-750 nm). This increased flash intensity is consistent with ablative vaporization of hypersonic fragments as the lower heat capacity of the monatomic argon as compared to residual air would be expected to generate higher gas temperatures in the post-shock gas flow around the hypersonic fragment. The higher gas temperature induced in argon provides more aggressive thermal ablation and excitation of the condensed phase metal.

S6 Integrated Wide-Band Emission

The previously discussed spectra are gated to evaluate emission characteristics at specific times during the flash, however, for selected impacts we collected long integration time (100 μs after impact) emission spectra over the wavelength range of 350-900nm using an Avantes AvaSpec compact spectrometer. The wide-band spectra of an Al/Al impact in 15 Torr air is shown in Fig. S8A, showing that the overall flash is dominated by Al atomic and Al-O combustion emission. There is negligent relative intensity emitted below 520 nm, so even at longer wavelengths where ambient gas species emission or continuum emission may be present, the aluminum emission processes constitute almost all the visual light emitted. When impacting a SS target (Fig. S8B), the total emission intensity is greater, allowing additional Al-O peaks to be observed, but aluminum combustion even further dominates the flash spectra.

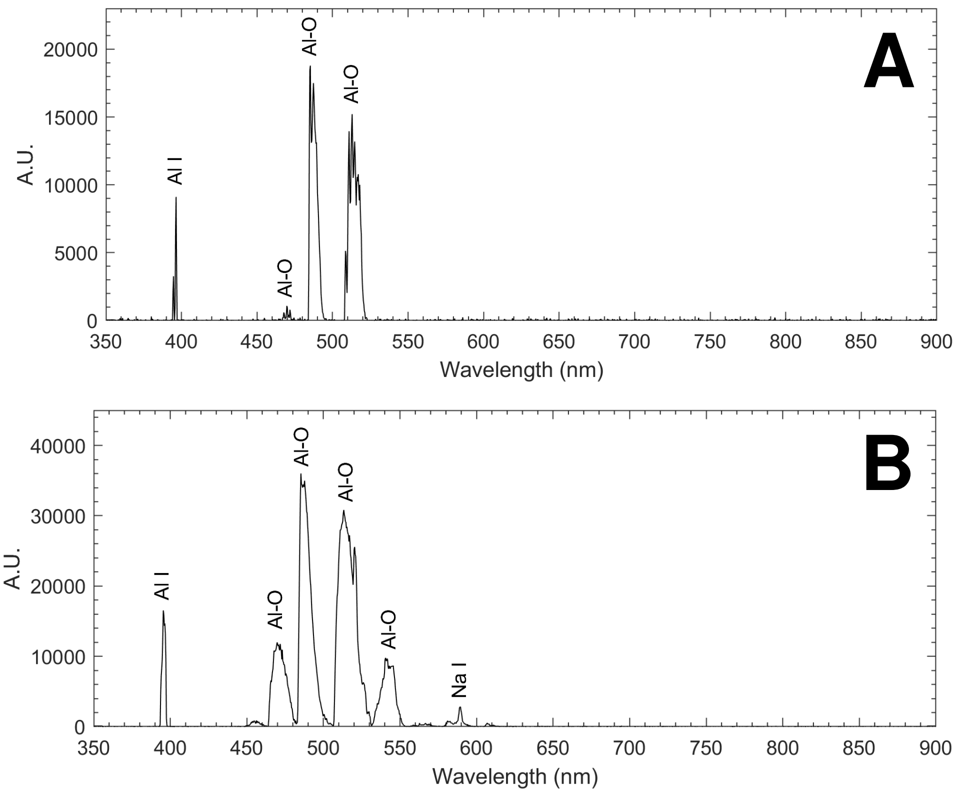

Supplementary Fig. S8: Wide-band long-exposure emission spectrum of 2024Al projectile impacting 6061Al target at 90° obliquity with normal velocity of 3.2 km/s in 15 Torr air (A) and 2024Al projectile impacting 304SS target at 90° obliquity with normal velocity of 3.1 km/s in 15 Torr air. Unlike the previous gated spectra, a 100 μs integration time (Avantes AvaSpec compact spectrometer) was used to observed the wide-band spectral characteristics of the entire flash emission. Optical window material limited minimum wavelength to $\sim$ 350 nm by transmission for this measurement.

Supplementary References

[1] K. Kurosawa, Y. Nagaoka, H. Senshu, K. Wada, S. Hasegawa, S. Sugita, and T. Matsui, "Dynamics of hypervelocity jetting during oblique impacts of spherical projectiles investigated via ultrafast imaging", Journal of Geophysical Research: Planets **120**, 1237–1251 (2015).

[2] A. M. Vickery, "The theory of jetting: application to the origin of tektites", Icarus **105**, 441–453 (1993).

[3] S. Sugita and P. H. Schultz, "Spectroscopic characterization of hypervelocity jetting: comparison with a standard theory", Journal of Geophysical Research: Planets **104**, 30825–30845 (1999).

[4] M. Zhaoxia, S. Anhua, L. Junling, L. Hai, L. Pei, and L. Sen, "Radiation mechanism analysis of hypervelocity impact ejecta cloud", International Journal of Impact Engineering **141**, 103560 (2020).

[5] L. S. H. Data and S. Marsh, *Los alamos series on dynamic material properties*, 1980.

[6] *Online materials information resource.*

[7] J. Walsh, R. Shreffler, and F. Willig, "Limiting conditions for jet formation in high velocity collisions", Journal of Applied Physics **24**, 349–359 (1953).

[8] V. A. Bronshten, *Physics of meteoric phenomena*, Vol. 22 (Springer Science & Business Media, 2012).

[9] T. J. Black and A. F. Cheviakov, "3drsp: matlab-based random sphere packing code in three dimensions", SoftwareX **18**, 101051 (2022).

[10] D. Grady, *Physics of shock and impact, volume 2* (IOP Publishing, 2017).

[11] G. H. Miller, "Jetting in oblique, asymmetric impacts", Icarus **134**, 163–175 (1998).

[12] B. C. Johnson, D. A. Minton, H. Melosh, and M. T. Zuber, "Impact jetting as the origin of chondrules", Nature **517**, 339–341 (2015).

[13] S. Wakita, B. C. Johnson, C. A. Denton, and T. M. Davison, "Jetting during oblique impacts of spherical impactors", Icarus **360**, 114365 (2021).
